# Limited Feasibility Study of Holographic Display Technology for Interprofessional Team Training

**DOI:** 10.3390/healthcare14050679

**Published:** 2026-03-07

**Authors:** Maria Bajwa, Melissa Morris, Wajeeha Brar Ghias, Adam Linzels

**Affiliations:** 1 Department of Health Professions Education, MGH Institute of Health Professions, Boston, MA 02129, USA; 2 Office of Innovation and Information Technology (OIIT), Nova Southeastern University, Fort Lauderdale, FL 33328, USA; mm2409@nova.edu (M.M.); linzels@nova.edu (A.L.); 3 Department of Leadership and Management Studies, Faculty of Contemporary Studies, National Defence University, Islamabad 44000, Pakistan; wajeeha@ndu.edu.pk

**Keywords:** healthcare simulation, interprofessional education, holographic display technology (HDT), TeamSTEPPS, interprofessional training, healthcare education, technology adoption in healthcare, healthcare workforce, healthcare professionals’ education, healthcare delivery, professionalism

## Abstract

**Highlights:**

**What are the main findings?**

- Healthcare professionals responded positively to holographic, team-based training, reporting improved perceptions of usefulness, support, and confidence in using the technology for learning.
- After training, participants showed stronger intentions to use holographic technology, supported by feedback describing engagement and relevance to real-world teamwork.

**What are the implications of the main findings?**

- Holographic, interprofessional training may support professional development that strengthens teamwork skills important for safe, effective healthcare delivery and patient care.
- These findings inform future studies exploring how immersive, distance simulation-based training can support healthcare workforce development and improve care quality over time.

**Abstract:**

**Background**: Immersive technologies are increasingly used to support interprofessional education and team training in healthcare. Holographic display technology (HDT) offers a novel approach for delivering distributed, simulation-based TeamSTEPPS training; however, evidence regarding its short-term feasibility remains limited. **Methods**: This mixed-methods pilot feasibility study examined the acceptability and limited efficacy (defined as learning satisfaction and self-reported gains) of HDT for interprofessional TeamSTEPPS-based team training across two geographically distributed campuses. Quantitative measures assessed changes in UTAUT-informed constructs, including attitude toward technology use (ATU) and behavioral intention (BI), while qualitative focus groups explored learner experiences and perceptions. **Results**: Of 64 participants, 47 consented to analysis. Quantitative analyses demonstrated post-training improvements in key technology-acceptance constructs, including significant gains in ATU and strengthened alignment between BI and UTAUT predictors. Qualitative findings reflected high learner engagement and perceived educational value, alongside practical considerations related to technical and instructional coordination. **Conclusions**: HDT was feasible for assessment of short-term foci of acceptability and perceived limited efficacy through the delivery of interprofessional TeamSTEPPS training, with observed gains in ATU and BI. These findings inform future assessment of long-term feasibility foci, including implementation studies examining the role of holographic simulation in advancing interprofessional education, healthcare workforce development, and the quality of healthcare delivery.

## 1. Introduction

The evolution of information and communication technologies has long enabled the delivery of healthcare education at a distance through simulation [1]. The COVID-19 pandemic further accelerated the adoption of e-learning, substantially expanding the use of simulation-based technologies in healthcare education [2]. Hence, healthcare educational facilities are investigating interactive means of technology-enhanced learning. Holographic display technology (HDT) is one such novel development that has the potential to revolutionize healthcare education [3], which allows healthcare professionals to learn in a much more engaging manner without compromising patient care [4]. However, despite multiple potential avenues to benefit healthcare and healthcare education, integrating HDT into healthcare education is cumbersome due to multiple organizational and individual-level barriers [4,5]. This pilot study aimed to assess the feasibility of HDT in the context of limited efficacy and acceptability among learners of various health professions to inform early adoption and future implementation decisions. For this purpose, a team training simulation using Team Strategies and Tools to Enhance Performance and Patient Safety (TeamSTEPPS) was conducted across two campuses where HDT delivered the content [6].

### 1.1. Overview of Holographic Display Technology (HDT)

Across educational and clinical settings, HDT has been applied in multiple contexts, with differences driven primarily by interface design and intended use. HDT enables three-dimensional (3D) projection of objects or individuals that supports interactive engagement and learner satisfaction [7]. In educational environments, institutions have adopted virtual anatomy tables or head-mounted displays to wear and conduct simulation in extended reality to enhance immersive learning [8,9,10]. In clinical settings, HDT has been used to support remote surgical applications and to facilitate patient interactions in infectious disease contexts while minimizing exposure risk [3]. HDT may also be used as an alternative to simulated patients, with the added advantage of live holographic projection of an individual portraying a patient [4]. Collectively, these applications demonstrate the versatility of HDT and provide a foundation for its extension to interprofessional team training, as examined in this study.

In the present study, the interface employed large-scale, screen-based holographic displays designed specifically to support interactive, team-based simulation and real-time collaboration across geographically distributed locations, a feature that distinguishes it from virtual anatomy tables and head-mounted display devices.

### 1.2. Feasibility Study

**Figure 1:**
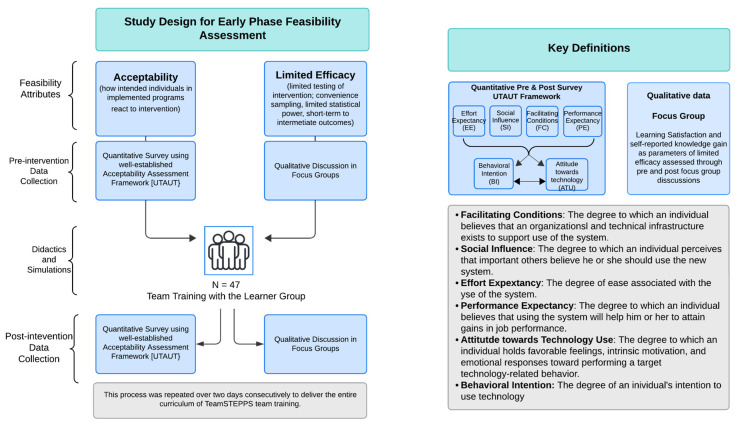
Flowchart outlining the early-phase feasibility assessment process for holographic display technology (HDT)-mediated interprofessional training.

While integrating technology-enhanced learning in healthcare education has great benefits, multiple parameters should be assessed to allow evidence-based application of simulation technology [7]. As trials are time-consuming and expensive to conduct, especially with the added cost of using simulation technology, a dedicated feasibility study for a particular intervention should be performed initially [11]. Among the eight focal points of an interventional feasibility study [12], acceptability and limited efficacy are particularly well suited to early-phase assessment; accordingly, these were selected as the focus of the present study (Figure 1). Consistent with the Bowen et al. framework for limited-efficacy testing using intermediate outcomes, learning satisfaction and self-reported knowledge gain were selected as proxy measures. For acceptability, four traits reported by the Unified Theory of Acceptance and Use of Technology (UTAUT) were studied [13,14]. See Appendix A for details of UTAUT. The other six foci, practicality, demand, implementation, adaptation, integration, and expansion, address feasibility over time and were, therefore, not considered for this target audience [12].

### 1.3. TeamSTEPPS

The Department of Defense (DoD) and the Agency for Healthcare Research and Quality (AHRQ) initiated the TeamSTEPPS project, and it has been ubiquitously used for team training in healthcare systems [6,15]. It comprises a patient-care framework with four skills: Communication, Leadership, Situation Monitoring, and Mutual Support. These skills are necessary to generate knowledge, attitude, and performance outcomes. The adequate bidirectional interplay of these skills and outcomes enhances the structure of a patient-care team [15]. TeamSTEPPS was chosen for HDT due to its universal applicability across healthcare systems and its routine inclusion in the university’s curriculum, as established by AHRQ [6]. Its integration required minimal disruption to the academic calendar, leveraging staff familiarity with the content and potential challenges of such professional development programs.

### 1.4. Ethical Considerations

The ethical review board of Nova Southeastern University deemed this study exempt (NSUIRB #2023-223). Written consent from the participants was obtained at the start of the training sessions. Data from 10 attendees who did not consent were excluded. Consenting participants’ data were stored in a secure, password-protected university cloud system, accessible only to MB and MM, with all identifying information removed to ensure anonymity.

## 2. Materials and Methods

### 2.1. Study Design

This pilot study used a mixed-methods, one-group pre–post educational intervention design to evaluate the feasibility of HDT for interprofessional team training [16]. (See Figure 1 for study design.) The quantitative component examined changes in UTAUT constructs from pre- and post-intervention, with these constructs operationalized as standardized indicators of learner acceptability rather than measures of educational outcomes, effectiveness, or efficiency consistent with feasibility study guidance [12]. Accordingly, quantitative analyses were intended to support feasibility signal detection and pattern identification, not hypothesis testing or causal inference. We analyzed the transcripts of focus group thematically to explore self-reported knowledge gain, learning experiences, and perceived impact on teamwork, yielding complementary, in-depth accounts aligned with the feasibility aims [17,18]. These qualitative data were used to contextualize acceptability and early indicators related to perceived learning outcomes and satisfaction towards team training, rather than to evaluate educational effectiveness or performance change.

### 2.2. Sampling and Recruitment

There were no specific sampling criteria as team training is provided as a routine professional development effort to all healthcare professional students and faculty throughout the year. Recruitment was done voluntarily through standard university advertisements for the event. All enrollees were invited to participate in this study. As a pilot feasibility study, the sample size was determined by the total number of trainees in the scheduled professional development program (convenience sampling) rather than a priori power analysis.

### 2.3. The HDT Simulation Setup

**Figure 2:**
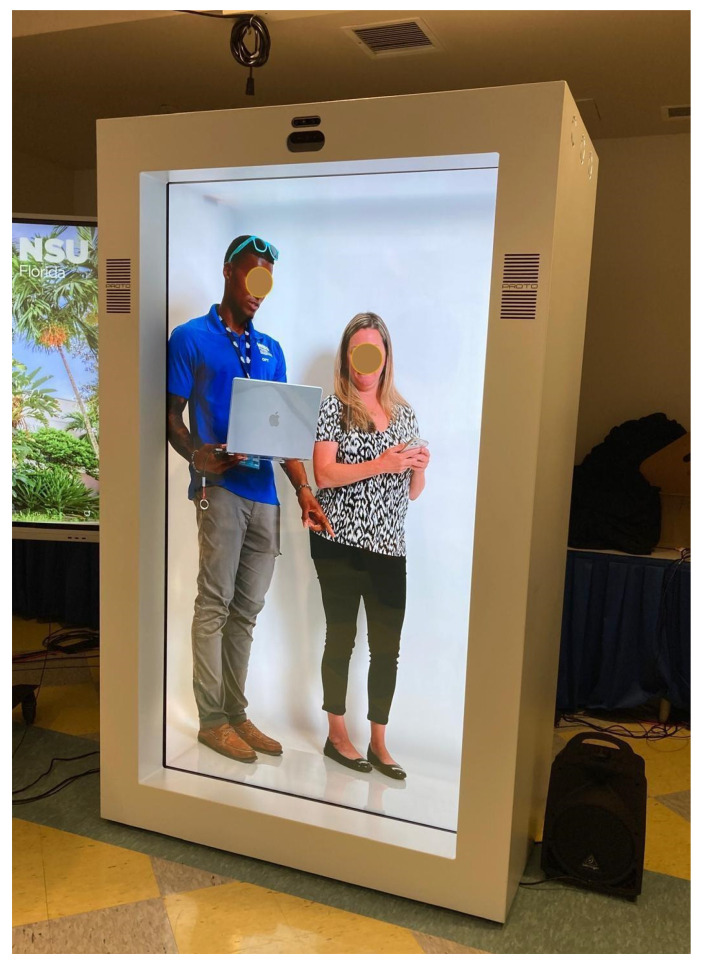
Simulated Participants as Viewed by the Learners. Viewed as life-sized, three-dimensional projection enabled by HDT while preserving visual presence and interaction fidelity.

The HDT is a screen-based system that uses volumetric 3D visualization, layered visual planes, and real-time interaction to create the illusion of depth and remote presence. The setup comprised two separate equipment configurations located at different sites: a streaming (broadcasting) unit and a viewing unit. The streaming (broadcasting) unit is equipped to capture and stream the live simulations with participants against a white backdrop. Participants are captured as real-life-like images to be displayed on the life-sized remote-viewing display unit, which creates a 3D holographic effect for viewers. Viewing units are standalone, rectangular box-shaped 3D systems that create a visual sensation of depth, tricking the viewer into seeing a 3D holographic image, as shown in Figure 2.

**Figure 3:**
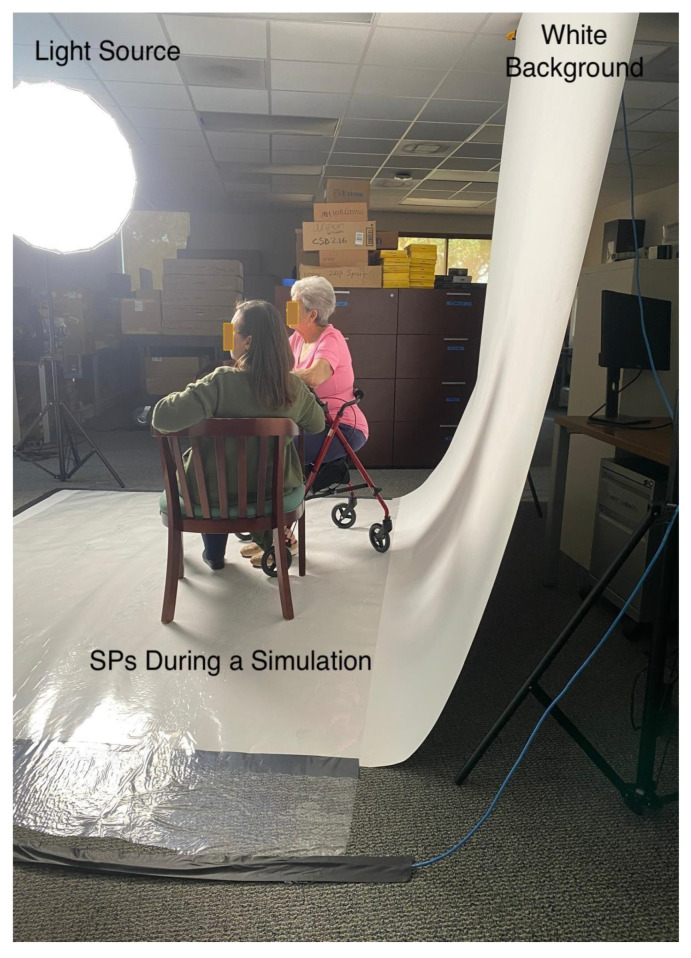
Simulated participants during the simulation as seen from the streaming area.

**Figure 4:**
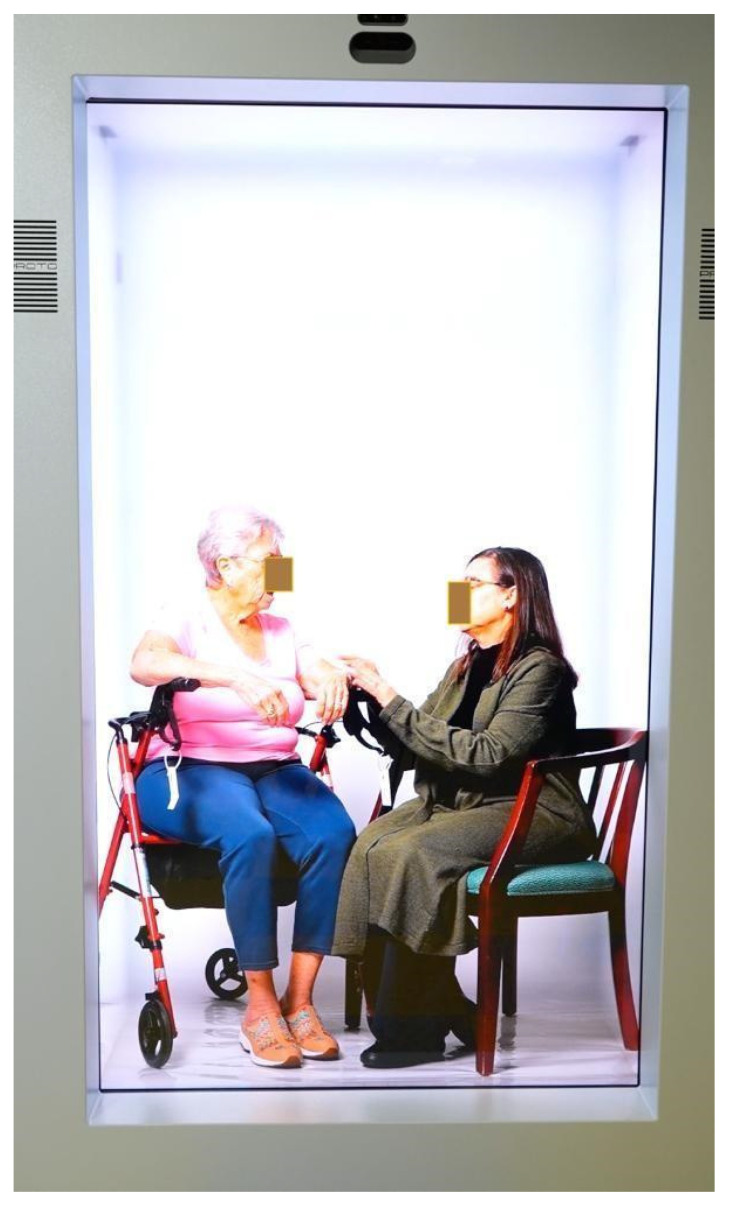
Simulated participants during the simulation as seen on the viewing unit.

The two-day intervention in this study was delivered across two campuses of a large university in the Southern United States, approximately 250 miles apart, connected via holographic display. Both campuses were equipped with the required HDT streaming (Figure 3) and viewing systems (Figure 4). Simulations were first conducted at one campus and streamed to the other, then repeated in reverse, with the second campus streaming and the first campus receiving. Audio–visual information technology technicians maintained these setups. The two campuses were additionally connected via videoconferencing as a contingency, with a supplementary camera providing audio and video support if needed.

The sequence of activities remained consistent across both days: a pre-activity survey, a didactic session, a simulated participant (SP)-based TeamSTEPPS simulation, and a post-activity survey, with the only planned variation being the simulation format (Day 1 prerecorded and delivered via HDT vs. Day 2 live holographic activity). See Appendix B for the training components and agenda. TeamSTEPPS team training curriculum was delivered over 2 days, using HDT in the following ways: (1) For maintaining communication between both campuses; (2) For delivering the pre-recorded scenario content at each campus; and (3) For allowing the participants to interact with the SP within the viewing holographic box. See Figure 4.

All simulations followed established best practices in healthcare simulations in accordance with published distance simulation guidelines [19,20]. Following the evidence-based practices, simulation scenarios were designed with the help of subject matter experts for the content and the information technology department for the technological specifications. Structured pre-briefing, SP-based activity, and debriefing were conducted for each simulation with the help of simulation-trained educators.

### 2.4. Data Collection and Management

Quantitative data were collected at four time points (Pre-T1, Post-T1, Pre-T2, and Post-T2) using surveys (see Appendix C) based on the UTAUT model [13]. UTAUT was selected as a validated and widely used framework for evaluating user acceptance of new technologies in health and education settings [14,21]. This approach was used to assess acceptability, a key construct of feasibility, particularly relevant for early-stage interventions when longer-term outcomes are not yet available. The UTAUT model is well-suited for evaluating responses to HDT in interprofessional team training as a professional development initiative.

Survey items were rated on a Likert scale (1 = strongly disagree to 5 = strongly agree) and assessed four core UTAUT constructs: performance expectancy (PE), effort expectancy (EE), social influence (SI), and facilitating conditions (FC). Composite scores were then calculated to derive ATU and BI, consistent with Venkatesh’s UTAUT framework [13]. Although multiple timepoints were analyzed, this report focuses on comparing Pre-T1 and Post-T2 to provide a clear summary of change over the full intervention period, aligning with feasibility study guidelines emphasizing pre–post comparisons.

Qualitative data on learner satisfaction, experience with HDT, and self-reported knowledge gain were collected through pre- and post-activity focus groups. (See Table 1 for the focus group prompts.) Focus group discussions were used to complement survey data by eliciting in-depth accounts of participants’ experiences, perceived value, engagement, and perceived impact on teamwork, thereby explaining and contextualizing the quantitative indicators of acceptability and limited efficacy. Fifteen attendees were randomly selected for these discussions; eight attended the pre-activity focus group, and 11 attended the post-activity focus group conducted at the conclusion of training. Focus group discussion data were thematically analyzed [17]. MB and MM familiarized themselves with data through multiple assessments of the recording and transcripts. Keywords and codes were identified, followed by categorizing the codes into themes. The themes were analyzed to develop a connection to UTAUT. By arranging numerous meetings, conflicts were resolved, and the data quality and validity were maintained. This enabled the development of theme definition, interpretation, and significance concerning the study’s aims [17]. Quantitative and qualitative findings were integrated through comparison of convergent and divergent patterns, using qualitative themes to explain or elaborate survey trends related to acceptability and limited efficacy.

## 3. Results

### 3.1. Demographics

Of the 64 learners who participated in the activity, data from 47 were included in the final analysis (five incomplete surveys; 12 non-consents). Analysis was focused on pre-training Day 1 (Pre-T1) and post-training Day 2 (Post-T2) to assess the overall intervention impact. Participants were predominantly female (n = 33, 70%) and English-speaking (n = 46, 98%), representing diverse professions, including Physical Therapy students (n = 25) and Nursing faculty/students (n = 10). The majority were aged 21–30 (n = 34) and reported limited prior experience with teamwork training. Detailed demographic breakdowns are provided in Appendix D.

### 3.2. Quantitative Results

Quantitative analyses examined acceptability and limited efficacy using UTAUT-informed measures, with ATU and BI serving as key outcome constructs. Data were collected at four time points (Pre-T1, Post-T1, Pre-T2, and Post-T2). For brevity, this manuscript reports Pearson correlation and Wilcoxon-ranked tests for pre- and post-intervention (Pre-T1 and Post-T2). Please see Appendix D and Appendix E for more details. 

#### 3.2.1. Pearson Correlations: Pre-T1 vs. Post-T2

At baseline (Pre-T1), Pearson correlations indicated positive associations between ATU and PE, EE, SI, and FC. These associations remained strong or increased by Post-T2. Notably, the correlations between ATU and both SI and FC were stronger at the end of Day 2 than at baseline, suggesting a closer alignment of social and contextual factors with attitudes toward holographic technology (Table 2).

#### 3.2.2. Change over Time: Wilcoxon Tests: Pre-T1 vs. Post-T2

Because ATU and BI scores were non-normally distributed and contained outliers, changes from pre-T1 to post-T2 were examined using Wilcoxon signed-rank tests. Parameters, SI, ATU, and BI showed significant improvement from Day 1 baseline to the end of Day 2, while PE, EE and FC demonstrated no significant change. Overall, results suggested improvement in participants attitudes, social perception and intention to use the system while other remained unchanged. (Table 3; Appendix E).

### 3.3. Qualitative Results

Qualitative themes derived through Braun and Clarke’s thematic analysis illustrate participants’ perceptions of HDT’s acceptability and its perceived impact on teamwork (limited efficacy).

#### 3.3.1. Pre-Activity Focus Group Results

The discussion revealed a complex interplay of excitement, apprehension, and speculation among participants regarding this technological innovation and team training. We identified four themes through thematic analysis, which provided insights into the multifaceted impact of integrating holography into learning environments. These included (1) Attitude Toward Technology, (2) Educational Technology Evolution, (3) Digital Teamwork Dynamics, and (4) Strategic and Practical Implications (See Appendix F and Appendix G for the quotations from the participants for pre- discussion).

**Attitudes toward technology.** Encompasses a spectrum of emotions and psychological responses; the codes consist of enthusiasm, apprehension, and curiosity for novelty. Participants discussed their perceptions in-depth, such as excitement and apprehension towards new technologies, their effect on morale, and the adoption of technology in healthcare education.

**Educational technology evolution.** Describes the evolution of technology and its integration into educational and training settings as perceived by the students. Individual codes included adding value, technology integration, and virtual versus hands-on training. Participants expressed their personal observations regarding the advancement and evolution of education owing to technology.

**Teamwork dynamics.** Emphasizes the influence of cutting-edge technology, like HDT, on interprofessional interaction and team building. Individual codes included communication, role clarity, leadership, and patient-centered care. Participants reinforced that core competencies, communication, role clarity, and leadership remain central, with technology serving as a resource rather than a replacement for team coordination.

**Strategic and practical implications.** Addresses the obstacles and challenges in implementing new technologies in educational settings and the strategies for navigating through them. Individual codes included technology in educational and patient-related care. Participants considered the implications of technology-enhanced learning, expressing concerns ranging from potential cognitive overload in a digital era to challenges in teaching practical skills like patient interaction.

#### 3.3.2. Post-Activity Focus Group Results

The HDT simulation activity garnered mixed reactions, highlighting the significant potential for educational innovation. We constructed the themes of (1) Technological Engagement, (2) Practical Concerns and Limitations, (3) Varied Effectiveness, and (4) Educational Impact (See Appendix F for the quotations from the participants for post-discussion).

**Engagement with Technology.** Captures learner interaction and immersion. Participants reported high engagement with the “live” holographic presence, though some suggested having increasing interactivity through branching “choose-your-own-adventure” scenarios.

**Practical Concerns and Limitations.** Includes the practicality, logistical, and financial concerns. Participants questioned the cost-effectiveness of HDT compared to standard video conferencing and noted technical distractions such as audio desynchronization and spatial constraints.

**Varied Effectiveness.** Captures the context-dependent utility and effectiveness of the HDT. Participants acknowledged that HDT provided value in cases where observation of full body language and surrounding environment plays a role in providing patient care. Others felt that HDT offered limited added value for less complex interactions.

**Educational Impact.** Captures the outcome of the educational session. Participants recognized HDT as a unique facilitator for distributed interprofessional education, providing a shared “space” for professionals that traditional online methods lack.

Table 4 summarizes pre- and post-activity qualitative themes, showing the evolution of learners’ thought processes. Learners found HDT engaging and educationally promising while simultaneously expressing concerns about preserving human connection, practical implementation challenges, and context-dependent effectiveness for teamwork training.

Quantitative improvements in PE, SI, FC, and attitudes toward HDT were aligned with qualitative themes of engagement and perceived educational value, particularly for interprofessional learning and communication-focused scenarios, while qualitative data also highlighted nuanced concerns about cost, technical limitations, and variable-added value compared with traditional methods that were not reflected in survey scores.

## 4. Discussion

This mixed-methods pilot feasibility study examined the acceptability and limited efficacy as a proxy indicator, defined as learning satisfaction and self-reported gains, of HDT for interprofessional TeamSTEPPS-based training. Rather than treating quantitative and qualitative findings as parallel evidence streams, their integration highlights how experiential factors shaped learner acceptance patterns observed across the intervention. For instance, the significant quantitative increase in Attitude (ATU) and Behavioral Intention (BI) was not merely a statistical artifact but directly corresponded to the qualitative narrative shift from initial uncertainty to recognized educational value. Specifically, these shifts in technology acceptance constructs appear to reflect learners’ transition from novelty-driven engagement to a more practical appraisal of HDT’s relevance.

**Figure 5:**
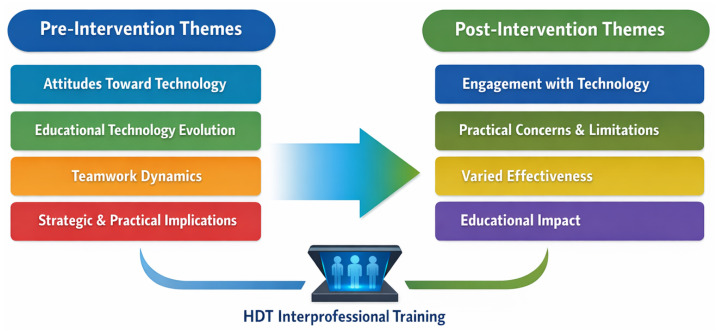
Evolution of Themes from Pre- to Post-Activity, highlighting the relationships and patterns that emerged from participant feedback.

The quantitative findings indicate that hands-on exposure to HDT altered how learners integrated perceptions of usefulness, ease of use, social influence, and contextual support into their intention to adopt the technology. Theoretically, this strengthening of the relationship between Behavioral Intention and predictors (PE, EE) suggests that hands-on experience moved learners from abstract expectations to concrete behavioral intentions. This pattern is consistent with early-stage adoption in immersive learning environments where acceptance emerges through experience rather than abstract expectation [22]. This pattern is interpreted as feasibility-relevant signals. Qualitative findings further contextualized these patterns by illustrating how learner perspectives evolved over time. Pre-intervention discussions emphasized technology integration, emotional reactions, and uncertainty, whereas post-intervention themes reflected greater focus on engagement, practical constraints, and educational fit (Figure 5). This thematic progression suggests a shift from pre-intervention themes (Attitudes Toward Technology, Education Technology Evolution, Teamwork Dynamics, and Strategic and Practical Implications) to post-intervention themes (Engagement with Technology, Practical Concerns and Limitations, Varied Effectiveness, and Educational Impact) (Figure 5).

Pre-activity discussion themes focused on technology integration and initial emotional responses, such as enthusiasm and apprehension. Before the intervention, the novelty of HDT strongly influenced learner perceptions, generating early engagement. Post-activity reflections shifted toward the significance of immersive learning, broader educational relevance, and interprofessional collaboration. One incidental but notable observation came from a neurodivergent participant who reported sustained engagement, an area warranting further investigation to explore potential benefits for diverse learners.

Participants also raised practical concerns after the activity, including technical challenges, human interactions, and cost–benefit considerations. This transition from initial excitement to critical evaluation highlights the value of feasibility studies in capturing real-world constraints. Ongoing concerns related to infrastructure may also explain why perceptions of facilitating conditions (FC) remained limited, despite overall improvements in technology acceptance. While the initial investment in HDT is higher than standard video conferencing, its potential to centralize expert instruction across distributed campuses without physical relocation may offer long-term efficiencies that offset the initial expense. These observations reflect early-phase feasibility, focused on acceptability and limited efficacy, prompting a longer-term assessment, including exploration of technological challenges [23].

While HDT shows promise in early-stage feasibility assessment, a single exposure cannot determine lasting behavioral change. Technology acceptance is frequently a non-linear trajectory, influenced by evolving user experience and contextual factors. Longitudinal evaluation is needed to assess sustained outcomes and explore broader feasibility domains, such as implementation, adaptation, and scalability [23].

Learner responses reflected a range of reactions, including both enthusiasm and initial hesitation. Some participants reported cognitive load, anxiety, or concern about reduced in-person interaction, responses commonly observed during the early adoption of novel educational technologies [22,24,25,26]. Consistent with established healthcare and distance simulation practices, the intervention incorporated structured pre-briefing and de-briefing to support psychological safety and learner engagement [19,20]. These concerns appeared to diminish as learners became more comfortable with the simulation environment, highlighting the role of evidence-based facilitation in supporting adaptation to new simulation modalities. As HDT develops, greater realism and broader simulation use could enhance its impact. Future studies should explore its acceptability across varied scenarios to guide effective integration into healthcare education.

These observations suggest that while HDT is accepted for skills training, it is most effective when implemented as a supplement to, rather than a replacement for, in-person mentorship to preserve the human connection learners value. Consistent with the pilot feasibility framework, these findings demonstrate the acceptability and limited efficacy of the intervention as defined by our study design, rather than implying confirmed instructional superiority.

### Strengths and Limitations

This study employed a mixed-methods design to evaluate the feasibility of holographic display technology (HDT) for interprofessional team training across geographically distinct sites. Integration within a TeamSTEPPS framework and collaboration among a multidisciplinary research team enhanced methodological rigor and relevance. Triangulation of quantitative UTAUT measures with qualitative thematic analysis strengthened interpretive validity and illustrated the capacity of HDT to support real-time interaction and debriefing across physical distance. Measurable shifts in acceptance and engagement suggest that even brief exposure to HDT may promote learner readiness for distributed team simulations.

Limitations include a modest, heterogeneous sample and lack of control over participants’ prior exposure to simulation or technology training, which may affect comparability. The heterogeneity in learner backgrounds and prior simulation exposure may have contributed to variability in engagement and perceptions of HDT. One confounding factor could be pre-recorded versus live sessions, where further exploration is warranted. Additionally, while recruitment targeted all program trainees, the voluntary nature of participation introduces the potential for self-selection bias, where individuals with a greater interest in technology may be overrepresented.

Technical challenges, such as spatial constraints, audio synchronization, and equipment costs, may limit generalizability and scalability. Accordingly, findings should be interpreted in terms of conceptual transferability of feasibility signals rather than statistical generalizability. The acceptability patterns and implementation considerations identified in this study reflect feasibility dynamics within a specific institutional, technological, and instructional context. While underlying principles related to learner engagement, technology acceptance, and implementation may inform other settings, their applicability will depend on local infrastructure, facilitation practices, and resource availability. Logistical requirements, including combining beaming and viewing in the same location, added complexity. Survey fatigue, due to repeated administration over 2 days, may have influenced data completeness and reduced response quality.

## 5. Conclusions

This mixed-methods feasibility study demonstrates that holographic display technology HDT is feasible for short-term interprofessional TeamSTEPPS training when evaluated using the feasibility foci of acceptability and limited efficacy. Observed improvements in ATU and BI, alongside learner reports of high engagement and perceived educational value, support HDT’s potential as an instructional modality. Implementation, however, required coordinated technical and instructional support, highlighting the importance of standardized facilitation.

Feasibility domains that assess performance over time, practicality, demand, implementation, adaptation, integration, and expansion were not examined in this study and, therefore, fall outside the scope of the current target audience. Future research should evaluate these additional feasibility foci to determine HDT’s scalability, long-term implementation, and sustained behavioral impact in advancing interprofessional education, workforce development, and healthcare delivery quality.

## Figures and Tables

**Table 1:** Focus group questions before and after the intervention.

Pre-Activity Learner Focus Group Questions	Post-Activity Learner Focus Group Questions
1. How are you feeling about this upcoming team training using holographic technology?	1. How are you feeling now that you have participated in the team training using holographic technology?
2. Are you feeling apprehensive or anxious, excited, or ecstatic about using this new technology? Please explain.	2. How was this experience for you?
3. What is the extent of your knowledge about Teamwork using TeamSTEPPS?	3. What did you learn today? Have you achieved all objectives, in your opinion?
4. In your opinion, what are the possible factors that can impact learning using holographic technology? (These could be negative or positive impacting factors.)	4. What problems or challenges did you encounter in this current session of team training using holographic technology that has or could have impacted your learning negatively?
5. What is the extent of your knowledge of teamwork?	5. What solutions can you think of to mitigate these challenges?

**Table 2:** Pearson correlations between UTAUT predictors, attitude toward technology use (ATU), and behavioral intentions (BI) at Pre-T1 and Post-T2.

Dependent Variable	Independent Variables	Pre-Training D1 r Value	Post Training D2 r Value
**Attitude toward Technology Use**	PE	0.628 **	0.633 **
	EE	0.585 **	0.591 **
	SI	0.544 **	0.692 **
	FC	0.416 **	0.616 **
**Behavior Intentions**	PE	−0.092	0.445 **
	EE	0.105	0.351 **
	SI	−0.029	0.401 **
	FC	−0.398 **	0.395 **
**Behavior Intentions association with Attitude toward Technology Use**		0.214	0.556 **

** Correlation is significant at the 0.01 level (2-tailed). Day 1 (D1), Day 2 (D2) Performance Expectancy (PE), Effort Expectancy (EE), Social Influence, Facilitating Conditions (FC), Attitude to Use Technology (ATU), and Behavior Intentions (BI).

**Table 3:** Wilcoxon Signed-Ranked Test Pre-Training D1 and Post-Training Day 2.

Paired Sample	Ranking	N	Mean Rank	Z Score	Sig (2-Tailed)
Performance Expectancy	Negative Ranks	7	6.43	−1.787	0.074
	Positive Ranks	3	3.33		
	Ties	37			
Effort Expectancy	Negative Ranks	4	2.50	−1.841	0.066
	Positive Ranks	0	0.00		
	Ties	43			
Social Influence	Negative Ranks	8	4.50	−2.524	0.012 *
	Positive Ranks	4	0.00		
	Ties	39			
Facilitating Conditions	Negative Ranks	4	2.50	−1.841	0.066
	Positive Ranks	0	0.00		
	Ties	43			
Attitude Toward Use	Negative Ranks	1	5.00	−2.689	0.007 *
	Positive Ranks	11	6.64		
	Ties	35			
Behavioral Intention	Negative Ranks	24	17.69	−3.005	0.003 **
	Positive Ranks	8	12.94		
	Ties	15			

Note: Wilcoxon Signed Rank Test was used to compare pre-training and post training scores. * *p* < 0.05, ** *p* < 0.01 indicate statistically significant difference

**Table 4:** Overview of Qualitative themes from pre- to post-activity focus groups.

Theme	Key Idea	Illustrative Quote
**Pre-activity Themes**		
Attitudes toward technology	Mixed excitement and apprehension about HDT and losing “human” elements.	*“Excited to see how it works, but I’m apprehensive.” (P3)* *“I feel like I’m kind of old-fashioned, I guess. I like regular in-person instructors that’s easier to interact with.” (P4)*
Educational technology evolution	HDT seen as part of a broader shift to diverse educational methods.	*“I am a former student. I actually took this from a different perspective. I’m really excited to see the hologram. I feel like it’s going to add to that variance of the educational methods,” (P2)*
Teamwork dynamics	Team coordination, communication, and leadership remain central despite new tech.	*“Pull in those resources instead of trying to control everything.” (P3)* *“When like a care team and knowing who to refer to, it’s important to remember that we’ve got this big push for patient-centered care.” (P4)*
Strategic/practical implications	Concerns about implementation, overload, and preserving patient-centered care.	*“The fear… is being cautious about it replacing a lived in-person human connection.” (P1)* *“So it’s the capacity to know your lane and when to advocate, reminding, remembering that at the end, it is the client care or the patient care in this case.” (P4)*
**Post-activity Themes**		
Technological engagement	Many found HDT engaging and wanted more interactive, branching scenarios.	*“It’d be fun, too, to use it as a choose-your-own-adventure kind of situation, where you’ve got multiple scenarios, they do like a short scene, and then they ask the audience, what should we do next? That way, it’s a little bit more interactive.” (P5)*
Practical concerns/limitations	Questions about cost, added value over Zoom, and technical/space constraints.	*“* *We’re trying to integrate it into the school. I feel that it’s long-term cost-effective as far as you’re not going to have to hire an additional person and have the structure at an alternative location. but for a couple $100,000?” (P1)*
Varied effectiveness	Viewed as highly useful in some communication/teamwork contexts, less so in others.	*“If you were teaching about conversational strategies and conflict resolution, it was good… otherwise, the holographic technology wasn’t required.” (P3)*
Educational impact	Potential to enhance interprofessional learning beyond traditional online methods.	*“The benefit would be in having all professionals in one place, teaching together.” (P4)*

## Data Availability

The data presented in this study are available on request from the corresponding author due to institutional policies governing data privacy and use.

## Appendix A. UTAUT Model Definitions

Root Constructs and Determinants of the UTAUT Model and their Definitions.

**Main Constructs/Determinants/Moderators**	**Root Construct Components**	**Definitions**
**Root Constructs/Determinants/**		
**1. Performance Expectancy** **(the strongest predictor of intention to use)**	U: Perceived Usefulness U1–U6	*The degree to which a person believes that using a particular system would enhance his or her job performance*
	EM: Extrinsic Motivation EM1–EM6	*The perception that users will want to perform an activity because it is perceived to be instrumental in achieving valued outcomes that are distinct from the activity itself, such as improved job performance, pay or promotions*
	JF: Job Fit JF1–JF6	*How the capabilities of a system enhance an individual’s job performance*
	RA: Relative Advantage RA1–RA5	*The degree to which using an innovation is perceived as being better than using its precursor*
	OE: Outcome Expectation OE1–OE7	*Relates to the consequences of the behavior*
**2. Effort Expectancy**	pEOU: Perceived Ease of Use EOU1–EOU7	*The degree to which a person believes that using a system would be free of effort*
	C: Complexity	*The degree to which a system is perceived as relatively difficult to understand and use*
	EOU: Ease of Use EOU1–EOU4	*The degree to which using an innovation is perceived as being difficult to use*
**3. Social influence**	SN: Subjective Norm SN1–SN2	*The person’s perception that most people who are important to him think he should or should not perform the behavior in question*
	SF: Social Factors SF1–SF4	*The individual’s internalization of the reference group’s subjective culture includes specific interpersonal agreements that the individual has made with others in specific social situations*
	I: Image I1–I3	*The degree to which the use of an innovation is perceived to enhance one’s image or status in one’s special system*
**4. Facilitating Conditions**	BC: Perceived Behavioral Control BC1–BC5	*Reflects perceptions of internal and external constraints on behavior and encompasses self-efficacy, resource-facilitating conditions, and technology-facilitating conditions*
	FC: Facilitating Conditions FC1–FC3	*Objective factors in the environment that observers agree make an act easy to do, including the provision of computer support*
	C: Compatibility C1–C3	*The degree to which an innovation is perceived as being consistent with existing values, needs, and experiences of potential adopters*
**Moderators impacting intentions to use directly or indirectly**		
**Age**		We are collecting age information as part of the demographics
**Gender**		We are collecting gender information as part of the demographics
**Experiences**		We are collecting previous technological experience information as part of the demographics
**Voluntariness**		*The degree to which the use of the technology is perceived as being optional or voluntary by the user, versus being mandatory*
**Attitude towards Technology**	A: Attitude towards Behavior A1–A4	*An individual’s positive or negative feelings about performing the target behavior*
	Intrinsic Motivation IM1–IM3	*The perception that users will want to perform an activity for no apparent reinforcement other than the process of performing the activity per se*
	Affect towards Use AU1–AU3	*Feelings of joy, elation, or pleasure, or depression, disgust, displeasure, or hate associated by an individual with a particular act*
	Affect Affect1–Affect5	*An individual’s liking of the behavior*
**Self-efficacy**	SE	*The belief in one’s capabilities to organize and execute the courses of action required to manage prospective situations.*
**Anxiety**	ANX	*Anxiety represents the level of unease or fear associated with using technology.*
**Behavioral Intention**	BI	*The degree of an individual’s intention to use the technology*
This table was informed by Venkatesh et al. (2003) [13] and Rouidi et al. (2022) [14].		

## Appendix B. TeamSTEPPS Training Components and Agenda

The TeamSTEPPS course will be delivered in a 2-day workshop using different educational strategies:

1. Didactics
  - Pre-session readings regarding
    - TeamSTEPPS
    - Technology
  - In-class lectures using PowerPoint
  - Introduction to the technology and demonstration
2. In-class interactive activities
  - Discussions on concepts
  - Case studies in small groups
  - Practice SBAR component of teamSTEPPS using Video stills and pictures
3. Simulation
  - Prebriefing
  - Video clip observation of a clinical case using holographic technology
  - Live role play observation of clinical team demonstrating the ‘huddle’ component of TeamSTEPPS
  - Debriefing
4. Evaluation of the simulation and workshop

**Agenda of the Workshop Days**

Day 1: 3 May 2023: 2:30–4:30 p.m. EST

2:00–2:20 Overview and pre-activity survey

2:20–2:40 Module 1—Introduction (Elizabeth Swann, PhD, ATC, FNAP)

2:40–3:05 Module 2—Team Structure (Kimberly Valenti, MS)

3:05–3:15 Bio Break

3:15–3:40 Module 3—Communication (Elizabeth Swann, PhD, ATC, FNAP)

3:40–3:55 SBAR Simulation

3:55–4:20 Module 4—Leading Teams (Maria Bajwa, MD, PhD, MSMS, RHIT)

4:20–4:30 Day 1—Post Survey

Day 2: 5 May 2022: 2:30–4:30 p.m. EST

2:00–2:15 Overview and pre-activity survey

2:15–2:45 Module 5—Situation Monitoring (Elizabeth Swann, PhD, ATC, FNAP)

2:45–3:15 Module 6—Mutual Support (Christine Kane EdD, MS, OTR/L)

3:15–3:25 Bio Break

3:25–3:50 Mutual Support (CUS) Simulation

3:50–4:10 Module 7—Summary (Live Hologram from Tampa)

4:10–4:30 Course Evaluation (Post Day 2 Evaluation)

## Appendix C. Quantitative (UTAUT) Survey Questions

The survey is set up on a five-point Likert scale, where one is strongly disagree, to five, which is strongly agree (Venkatesh et al., 2003) [13].

Performance Expectancy

U2: Using this system would improve my job performance

U6: I would find the system useful in my job.

RA1: Using the system enables me to accomplish tasks more quickly.

RA2: Using the system improves the quality of my work

RA5: Using the system increases my productivity.

OE2: If I use this system, I will spend less time on routine job tasks

OE7: If I use the system, I will increase my chances of getting a raise

Effort Expectancy

EOU3: My interaction with the system would be clear and understandable.

EOU5: It would be easy for me to become skillful at using the system.

EOU6: I would find the system easy to use.

EU4: Learning to operate the system is easy for me

C1: Using this system takes too much time from my normal duties

Social Influence

SN1: People who influence my behavior think that I should use the system.

SN2: People who are important to me think that I should use the system.

SF2: The senior management of this business has been helpful in using the system.

SF4: In general, the organization has supported the use of the system

I3: Having the system is a status symbol in my organization

Facilitating Conditions

PBC2: I have the resources necessary to use the system

PBC3: I have the knowledge necessary to use the system

PBC5: The system is not compatible with other systems [techniques and technologies] I use

FC3: A specific person (or group) is available to assist with system difficulties.

C2: I think that using the system fits well with the way I like to work

Attitude toward Using Technology

Al: Using the system is a good idea.

AF1: The system makes work more interesting.

AF2: Working with the system is fun.

Affect1: I like working with the h-tech system.

Self-Efficacy

I could complete a job or task using the system…

SE1: If there was no one around to tell me what to do as I go.

SE4: If I could call someone for help if I got stuck.

SE6: If I had a lot of time to complete the job for which the software was provided.

SE7: If I had just the built-in help facility for assistance.

Anxiety

ANX1: I feel apprehensive about using the system.

ANX2: It scares me to think that I could lose a lot of information by hitting the wrong key.

ANX3: I hesitate to use the system for fear of making mistakes I cannot correct.

ANX4: The system is somewhat intimidating to me.

Behavioral Intention

BI1: I intend to use the system in the next months.

B12: I predict I would use the system in the next months.

B13: I plan to use the system in the next months.

## Appendix D. Demographic Information of the Participants

Demographic Information of the Participants.

Total Learners = 47			
**Professions**	**Number**	**Profession**	**Number**
Physical Therapy Students	25	Nurse Practitioner	1
Mental health counselor and educator	1	Occupational Therapist	1
Administration	1	Emergency Medical Services	1
Health Informatics and Physical Therapy Assistant	2	Lawyer	1
Osteopathic Medicine	1	Faculty and Students	10
Nursing	2	PGY-1 Pharmacy Resident	1
**Sex**	**Number**	**Language**	**Number**
Female-0	33	English	46
Male-1	14	Other	1
**Age**	**Number**	**Age**	**Number**
21–30	34	51–60	2
31–40	5	61–70	2
41–50	3	71–80	1
**Year of Experience**	**Number**	**Year of Experience**	**Number**
0	36	3	1
1	2	4	0
2	1	5	7
**Teamwork Training (Years)**	**Number**	**Teamwork Training (Years)**	**Number**
0	31	3	0
1	11	4	3
2	2	5	0
**TeamSTEPPS Training (Years)**	**Number**	**TeamSTEPPS Training (Years)**	**Number**
0	38	3	2
1	3	4	4
2	0	5	0

## Appendix E. Complete Wilcoxon Signed-Ranked Test

Detailed table of the Wilcoxon Signed- Ranked Test from pre-activity to post-activity

**Test Statistics**						
	PEPTR2-PETR2	EEPTR2-EETR2	SIPTR2-SITR2	FCPTR2-FCTR2	ATPTR2-ATTR2	BIPTR2-BITR2
Z	−1.787 ^b^	−1.841 ^b^	−2.524 ^b^	−1.841 ^b^	−2.689 ^c^	−3.005 ^b^
Asymp. Sig. (2-tailed)	0.074	0.066	0.012	0.066	0.007	0.003
a. Wilcoxon Signed Ranks Test, b. Based on positive ranks, c. Based on negative ranks.						

**Ranks**					
		**N**	**Mean Rank**	**Sum of Ranks**	
PEPTR2-PETR2	Negative Ranks	7 ^a^	6.43	45.00	a. PEPTR2 < PETR2
	Positive Ranks	3 ^b^	3.33	10.00	b. PEPTR2 > PETR2
	Ties	37 ^c^			c. PEPTR2 = PETR2
EEPTR2-EETR2	Negative Ranks	4 ^a^	2.50	10.00	a. EEPTR2 < EETR2
	Positive Ranks	0 ^b^	0.00	0.00	b. EEPTR2 > EETR2
	Ties	43 ^c^			c. EEPTR2 = EETR2
SIPTR2-SITR2	Negative Ranks	8 ^a^	4.50	36.00	a. SIPTR2 < SITR2
	Positive Ranks	0 ^b^	0.00	0.00	b. SIPTR2 > SITR2
	Ties	39 ^c^			c. SIPTR2 = SITR2
FCPTR2-FCTR2	Negative Ranks	4 ^a^	2.50	10.00	a. FCPTR2 < FCTR2
	Positive Ranks	0 ^b^	0.00	0.00	b. FCPTR2 > FCTR2
	Ties	43 ^c^			c. FCPTR2 = FCTR2
ATPTR2-ATTR2	Negative Ranks	1 ^a^	5.00	5.00	a. ATPTR2 < ATTR2
	Positive Ranks	11 ^b^	6.64	73.00	b. ATPTR2 > ATTR2
	Ties	35 ^c^			c. ATPTR2 = ATTR2
BIPTR2-BITR2	Negative Ranks	24 ^a^	17.69	424.50	a. BIPTR2 < BITR2
	Positive Ranks	8 ^b^	12.94	103.50	b. BIPTR2 > BITR2
	Ties	15 ^c^			c. BIPTR2 = BITR2

## Appendix F. Pre-Activity Focus Group Detailed Quotations

Themes were constructed from pre-activity focus groups.

**Themes**	**Quotations**
**Attitudes Towards Technology**	- *I’m really excited to see the hologram (P1).* - *It makes me kind of apprehensive about this (P3).* - *I think it’s interesting, holographic anything… the technology, I think, is really cool (P5).* - *Whenever we’re adding a new piece of technology, there’s always the novelty factor. And especially for something like holograms and VR, it’s very hollow-deck-y, it’s very sci-fi (P2).*
**Education Technology Evolution**	- *I think that technology, when it is additive to our experience, is wonderful (P4).* - *I have to admit that my first thought was, oh, what a claustrophobic experience to have to be in that box with someone else…. Maybe I’m not familiar with how this is going to work. But after hearing some of the feedback from the osteopathic medicine program when they integrated some more of this technology without having a professor present or using the cadaver tables.… I think there’s a benefit to us (P3).* - *It’s like you get to actually see the body language, which is really important in healthcare. And it can sometimes affect the way you respond (P2).* - *I think it’s great as far as learning things like patient interviewing techniques and kind of getting an initial sense of what sick versus not sick is and developing how to interact with patients. But I don’t really feel, and this is my personal opinion, that there is a true replacement for putting your hands on a patient or doing an activity (P1).*
**Teamwork Dynamics**	- *I feel like it’s so important, the communication aspect of knowing how to communicate and hand off a patient correctly, so that everyone is informed on a team, because that’s the only way to get the most effective treatment and care (P3).* - *I think a lot of that goes to understanding the role of other people on your team and recognizing that that leader might not be a subject matter expert in something. And whoever is in a leadership or a coordinator position can delegate the task to somebody who is more knowledgeable about it (P3).* - *And when you come into a group where everybody may be a leader in their field when they come together, sometimes it’s hard. You know, if someone takes that lead real quick, then it might be easy. But sometimes I feel like people don’t want to step on other people’s toes (P2).* - *It’s important that as providers, we’re communicating with the other members of the care team and offering our resources and then educating the patient about their options as well (P1).*
**Strategic and Practical Implications**	- *Just the excitement of using a technology like that, people are gonna be using up a little bit of their mental bandwidth. And so that can leave less mental bandwidth available for what we’re actually supposed to be learning about and listening to what your team members are saying and things like that (P2).* - *It’s hard to really design effective training that’s going to make them able to use the tool (P5).* - *I have a little bit of VR knowledge. But I’ve never had an opportunity to sort of put it all together (P3).* - *This [technology] is kinda tied into telemedicine. And maybe I shouldn’t be categorizing it with similar things to telemedicine. But what I’ve seen in telemedicine [is] that patients aren’t getting the care they really should be with an in-person visit. I’m seeing psych patients bounce back within 24 h because they aren’t having a true personal assessment done (P2).”* - *So, for the most part, we are virtual, and we’re kind of learning on our own. I feel like it kind of gives us, further, a way of being able to practice hands-on (P2).*
Note: This table presents themes and representative quotations gathered from participants during the pre-activity focus group. Themes were derived from responses to questions about initial expectations and perceived readiness for the activity.	

## Appendix G. Post-Activity Focus Group Detailed Quotations

Themes were constructed from the discussion with the focus group participants after the activity.

**Themes**	**Quotations**
**Engagement with Technology**	- *I think it’s really cool. I especially liked how he was in the box like avatars are on the screen, and how you kind of have someone in the box, and someone actually be outside of it and like communicating back and forth.” (P4)* - *I am dyslexic…and my ADHD kicks in using Zoom…Today, with the hologram, you know, I was able to see the motion and the body movement, and that was able to keep my attention a little more. However, I do understand everyone else’s voice about, you know, is it worth all the money to have that just for, you know, a select couple of students. (P2)* - *I think I learned better with the simulation, with the interactive capabilities of it. (P1)*
**Practical Concerns and Limitations**	- *I don’t really see what difference the hologram provides outside of video conferencing.* - *It seems that it is about on par with a much larger price tag at this point. (P3).* - *When they are in the box, but the audio isn’t with the actual picture…it just became a distraction for me. (P1).* - *We’re implementing something that’s very much so less expensive using Zoom or another video conferencing and equipment. (P2)*
**Varied Effectiveness**	- *The main difference between the hologram box and Zoom is that when you are on Zoom, it’s just your head… if it were a scenario that involved some full-body movement or examination, I definitely think the hologram box would be an advantage. (P4).* - *I don’t think the intention of the hologram matched its presentation. It just didn’t seem like it was required. (P2).*
**Educational Impact**	- *If they had more standard practices, it would work better. However, I already kind of use these same strategies just in a different format, just from experience with working with different environments (P5).* - *It teaches us not to be just tunnel-visioned and stay in our lane, and that we can see more things from a global perspective. (P3).* - *This proves that interprofessional collaboration and practice really work… Imagine a country without easy access to healthcare providers—how do you deliver care in a 3D way? …this approach to be huge and incredibly helpful. (P2).*
Note: This table presents themes and illustrative quotations from the post-activity focus group, capturing participants’ reflections on their experiences and perceived outcomes following the activity.	
